# Biofilms Comprise a Component of the Annual Cycle of Vibrio cholerae in the Bay of Bengal Estuary

**DOI:** 10.1128/mBio.00483-18

**Published:** 2018-04-17

**Authors:** Marzia Sultana, Suraia Nusrin, Nur A. Hasan, Abdus Sadique, Kabir U. Ahmed, Atiqul Islam, Anwar Hossain, Ira Longini, Azhar Nizam, Anwar Huq, Abul K. Siddique, David A. Sack, Richard B. Sack, Rita R. Colwell, Munirul Alam

**Affiliations:** a International Centre for Diarrhoeal Disease Research, Bangladesh, Dhaka, Bangladesh; b Emory University, Atlanta, Georgia, USA; c Department of Microbiology, Dhaka University, Dhaka, Bangladesh; d University of Florida, Gainesville, Florida, USA; e Maryland Pathogen Research Institute, University of Maryland, College Park, Maryland, USA; f Johns Hopkins Bloomberg School of Public Health, Baltimore, Maryland, USA; g University of Maryland Institute for Advanced Computer Studies, College Park, Maryland, USA; University of Nebraska-Lincoln

**Keywords:** *Vibrio cholerae*, biofilms, cholera

## Abstract

Vibrio cholerae, an estuarine bacterium, is the causative agent of cholera, a severe diarrheal disease that demonstrates seasonal incidence in Bangladesh. In an extensive study of V. cholerae occurrence in a natural aquatic environment, water and plankton samples were collected biweekly between December 2005 and November 2006 from Mathbaria, an estuarine village of Bangladesh near the mangrove forests of the Sundarbans. Toxigenic V. cholerae exhibited two seasonal growth peaks, one in spring (March to May) and another in autumn (September to November), corresponding to the two annual seasonal outbreaks of cholera in this region. The total numbers of bacteria determined by heterotrophic plate count (HPC), representing culturable bacteria, accounted for 1% to 2.7% of the total numbers obtained using acridine orange direct counting (AODC). The highest bacterial culture counts, including toxigenic V. cholerae, were recorded in the spring. The direct fluorescent antibody (DFA) assay was used to detect V. cholerae O1 cells throughout the year, as free-living cells, within clusters, or in association with plankton. V. cholerae O1 varied significantly in morphology, appearing as distinctly rod-shaped cells in the spring months, while small coccoid cells within thick clusters of biofilm were observed during interepidemic periods of the year, notably during the winter months. Toxigenic V. cholerae O1 was culturable in natural water during the spring when the temperature rose sharply. The results of this study confirmed biofilms to be a means of persistence for bacteria and an integral component of the annual life cycle of toxigenic V. cholerae in the estuarine environment of Bangladesh.

## IMPORTANCE

Vibrio cholerae, the causative agent of cholera, is autochthonous in the estuarine aquatic environment. This study describes morphological changes in naturally occurring V. cholerae O1 in the estuarine environment of Mathbaria, where the bacterium is culturable when the water temperature rises and is observable predominantly as distinct rods and dividing cells. In the spring and fall, these morphological changes coincide with the two seasonal peaks of endemic cholera in Bangladesh. V. cholerae O1 cells are predominantly coccoid within biofilms but are rod shaped as free-living cells and when attached to plankton or to particulate matter in interepidemic periods of the year. It is concluded that biofilms represent a stage of the annual life cycle of V. cholerae O1, the causative agent of cholera in Bangladesh.

## INTRODUCTION

Bacteria in a natural ecosystem manifest morphological changes during different stages of their life cycle, from actively growing or culturable cells to dormant or nonculturable cells (1). Bacteria typically will be actively growing in laboratory culture and when in a host, as long as nutrients are adequate to support growth and reproduction. However, when in an environment where the nutrient concentration is below that required for growth and multiplication, the bacteria will adapt, employing a variety of mechanisms (2). For example, entry into a nonculturable or dormant state has been shown to occur when environmental conditions are unfavorable, e.g., starvation conditions, temperature extremes, changes in salt concentration such as occur during tidal flow of seawater, fluctuations in oxygen concentration, or exposure to light (3, 4). The microbial population in the natural environment has been shown to include significant numbers of bacteria that cannot be cultured in the laboratory using routine bacteriologic media and methods (5). It is also important to note that nonculturable cells retain metabolic activity that is detectable by various methods. Furthermore, both pathogenic and nonpathogenic strains of a species can coexist in a bacterial community, persisting either in the culturable state, as nonculturable cells, or both (5). Many species of bacteria pathogenic for humans have been shown to enter the nonculturable state, including *Campylobacter* spp., Escherichia coli (including enterohemorrhagic *E. coli* [EHEC]), Francisella tularensis, Helicobacter pylori, Legionella pneumophila, Listeria monocytogenes, Mycobacterium tuberculosis, Pseudomonas aeruginosa, several *Salmonella* and *Shigella* spp., Vibrio cholerae, Vibrio parahaemolyticus, and Vibrio vulnificus (1, 4).

In the aquatic environment, nonculturable bacteria have frequently been observed either attached to various substrates, including sediment particles, marine snow, fecal and silt particles in the water column, zooplankton and phytoplankton, or as free-living single or aggregated cells (usually in a biofilm) in the water column (6). The biofilm is generally described as an assemblage of microbial cells enclosed in a matrix of primarily polysaccharide material that allows the cells to remain attached, comprising consortia (7). Such assemblages or consortia can be composed of a population developing from a single species or a community derived from multiple microbial species. Clusters of biofilms can develop on various abiotic and biotic surfaces in the aquatic environment (8), developing in multiple layers of cells and eventually producing three-dimensional structures containing water channels through which nutrients diffuse in and waste products diffuse out (9, 10). Under favorable environmental conditions, the biofilm-associated bacteria detach from the biofilm matrix and disperse as actively growing (culturable) cells (11).

V. cholerae, the causative agent of cholera, is autochthonous to the estuarine environment, and interestingly, epidemiological data show that endemic cholera in Bangladesh usually emerges first in coastal villages, spreading inland. To date, the bulk of research and interventions carried out on cholera in Bangladesh have been done mainly in the freshwater ecosystem of Dhaka and Matlab, two inland sites of cholera endemicity located 350 km from the Bay of Bengal estuary. Thus, it is important to determine the mechanism(s) used by toxigenic V. cholerae to thrive in the aquatic environment during interepidemic periods and those factors determining seasonal cycles of endemic cholera. The aim of this study, therefore, was to examine the annual cycle of toxigenic V. cholerae in the natural environment and the mechanism of its response to seasonal climate changes characteristic of the estuarine ecosystem in the Bay of Bengal, Bangladesh.

## RESULTS

### Seasonal bacterial growth response and isolation and detection of toxigenic V. cholerae in the Bay of Bengal estuary.

**FIG 1:**
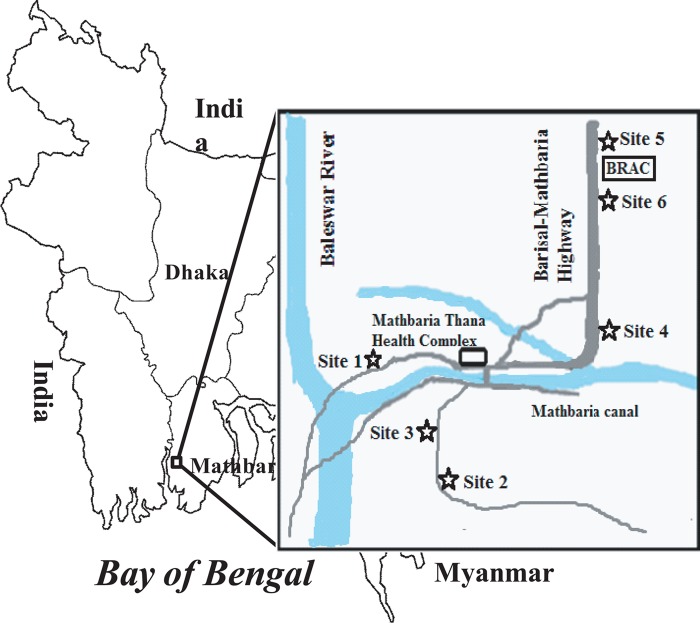
Map of Bangladesh showing the six sampling sites (stars) located in Mathbaria, a coastal village near the Bay of Bengal.

**FIG 2:**
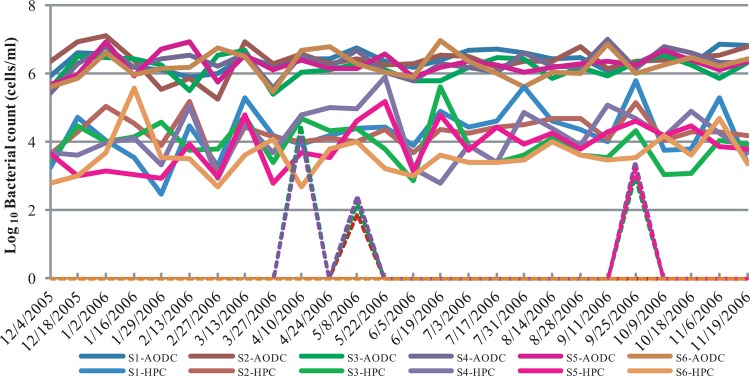
Total direct (AODC), culturable bacterial (HPC), and culturable V. cholerae O1 (VCO1) counts of water samples collected from six pond sites (S1 to S6) of the estuarine environment of Bangladesh between December 2005 and November 2006.

The study sites from where the water samples were collected are shown in Fig. 1. The results of the acridine orange direct counting (AODC) of total bacteria, total culturable bacterial counts measured as CFU determined by heterotrophic plate counting (HPC), and total counts of culturable V. cholerae O1 obtained from these samples were analyzed statistically. The Log_10_-transformed bacterial counts are presented in Fig. 2. As expected, the AODC counts were consistently higher than culture counts (HPC), by approximately 2 to 3 log. Peaks in culturable V. cholerae O1 counts obtained using conventional plate culture were observed in April to May and again in September, as shown by the results in Fig. 2. As shown by the results in Table 1, actively growing toxigenic V. cholerae O1 was highest (23.8%) in water samples collected during the spring months, when the first peak of the annual seasonal cholera begins in Bangladesh (12–15). Actively growing toxigenic V. cholerae was also found in the fall, but at a low frequency (7.1%), and the bacterium was not found active during the interepidemic periods of the year. Direct detection of toxigenic V. cholerae O1 from water was made by PCR targeting the genes *rfbO1*, encoding the surface O antigen of V. cholerae O1, and *ctxA*, encoding subunit A of cholera toxin (CT). PCR assays detected the genes *rfbO1* and *ctxA* from the highest number of water samples, accounting for 23.8% and 26.2%, respectively, in the spring, coinciding with the seasonal growth response of toxigenic V. cholerae O1 occurring naturally. These genes were also detected by PCR from surface water during and between the two defined seasons of cholera, but at lower frequencies (Table 1). These results suggest the consistent presence of the bacterium, either as actively growing or as dormant cells in the so-called nonculturable state, in the estuarine environment (Table 1) (1, 16, 17).

**TABLE 1:** Isolation and detection of toxigenic V. cholerae from water samples collected at Mathbaria, a region of Bangladesh where cholera is endemic, by culture and PCR methods

Season	No. of samples	No. (%) of samples with V. cholerae O1		
		Isolated by culture (%)	Detected by PCR of:	
			*rfbO1* (%)	*ctxA*(%)
Winter	36	0	3 (8.3)	4 (11.1)
Spring	42	10 (23.8)	10 (23.8)	11 (26.2)
Monsoon	36	0	2 (5.6)	2 (5.6)
Fall	42	3 (7.1)	3 (7.1)	3 (7.1)

**FIG 3:**
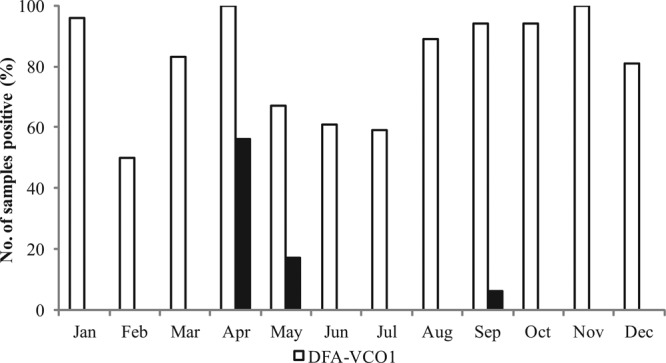
Percentages of samples positive for V. cholerae O1 (VCO1) determined by DFA and culture in water samples collected from the estuarine environment of Bangladesh between December 2005 and November 2006.

Based on the direct fluorescent antibody (DFA) results, V. cholerae O1 was present in the water throughout the year, with the number of positive samples varying from 50 to 100% (Fig. 2). The lowest percentage of samples testing positive for V. cholerae O1 by DFA occurred during February, whereas the highest percentages occurred during April and November (Fig. 2). Two annual peaks of culturable V. cholerae O1 were observed at four of the six study sites in Mathbaria, the first being in April and May and the second in September (Fig. 2 and 3). It is important to note that the total bacterial counts (AODC) in the water samples, including V. cholerae O1, exhibited two seasonal growth peaks, namely, in the spring (March to May) and fall (September to November), corresponding to the two well-characterized seasonal peaks of cholera in Bangladesh (12–15).

### Bacterial culturability and morphology related to season.

The culturable bacterial counts (HPC) of the water samples collected at the Mathbaria sites near the Bay of Bengal varied from 1% to 2.7% of the total bacterial count (AODC) (Table 2), and the proportion of culturable V. cholerae O1 similarly varied from 0 to 25.62% of the DFA counts, depending on season of the year (Table 3). The highest culturable V. cholerae O1 and culturable bacterial counts were obtained in the spring (25.62% of the DFA counts and 2.7% of the AODC counts, respectively), and the lowest in the fall season (0.03% of the DFA and 1% of the AODC count) (Tables 2 and 3 and Fig. 3). During the winter and monsoon months, V. cholerae O1 was not able to be cultured and the bacterial culture counts were reduced (1.4% and 1.3% of the AODC counts) (Tables 2 and 3 and Fig. 3).

**TABLE 2:** Counts of culturable heterotrophic bacteria for water samples collected from the coast of Bangladesh during different seasons of the year

Season	Months	Bacterial count (mean ± SD [min-max])		% of bacteria culturable
		Total (AODC) (cells/ml)	Culturable (HPC) (CFU/ml)	
Winter	December, January, February	2.7 × 10^6^ ± 2.2 × 10^6^ (1.8 × 10^5^–1.3 ×10^7^)	3.9 × 10^4^ ± 9.9 × 10^4^ (3.0 × 10^2^–8.0 ×10^5^)	1.4
Spring	March, April, May	2.7 × 10^6^ ± 1.8 × 10^6^ (2.5 × 10^5^–8.5 ×10^6^)	7.3 × 10^4^ ± 1.8 × 10^5^ (5.0 × 10^2^–8.0 ×10^5^)	2.7
Monsoon	June, July, August	2.3 × 10^6^ ± 1.7 × 10^6^ (4.3 × 10^5^–9.5 ×10^6^)	3.1 × 10^4^ ± 6.5 × 10^4^ (6.0 × 10^2^–4.0 ×10^5^)	1.3
Fall	September, October, November	3.1 × 10^6^ ± 2.1 × 10^6^ (7.3 × 10^5^–9.8 ×10^6^)	2.9 × 10^4^ ± 4.3 × 10^4^ (1.1 × 10^3^–2.0 ×10^5^)	1

**TABLE 3:** Culturability and morphology of V. cholerae O1 in water samples collected from the estuarine environment of Bangladesh during different seasons of the year

Season	Months	V. cholerae O1 count (mean ± SD [min-max]) by:		% of culturable V. cholerae O1	No. of samples with cells with indicated morphology/total no. of samples		
		DFA (cells/ml)	Culture (CFU/ml)		Curved rods, dividing rods, and thin biofilms	Coccoid, short rods, and thick biofilms	Other
Winter	December, January, February	7.2 × 10^3^ ± 1.7 × 10^4^ (0–7.5 × 10^4^)	0	0	0/42	41/42	1/42
Spring	March, April, May	1.4 × 10^5^ ± 4.0 × 10^5^ (0–1.5 × 10^6^)	3.5 × 10^4^ ± 1.9 × 10^5^ (0–1.2 × 10^6^)	25.62	30/36	0/36	6/36
Monsoon	June, July, August	1.3 × 10^5^ ± 2.8 × 10^5^ (0–1.0 × 10^6^)	0	0	0/42	34/42	8/42
Fall	September, October, November	3.4 × 10^5^ ± 5.5 × 10^5^ (2.5 × 10^1^–2.4 × 10^6^)	1.11 × 10^2^ ± 3.98 × 10^2^ (0–2.0 × 10^3^)	0.03	36/36	0/36	0/36

**FIG 4:**
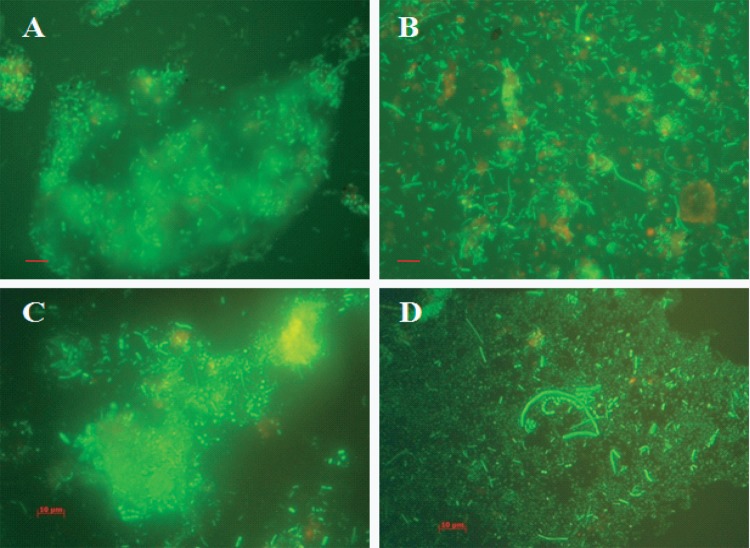
Micrographs of acridine orange-stained microbial communities occurring in the natural estuarine aquatic ecosystem of the mangrove forest, Sundarbans, Bay of Bengal, Bangladesh, where cholera is endemic. The micrographs provide *in situ* evidence of free-living bacteria in a natural aquatic ecosystem assembling and transforming into smaller and coccoid cells in the spring and fall (B and D) and forming biofilm consortia during the winter and monsoon months of the year (A and C). Scale bars in red indicate 10 µm.

Bacteria are observed as green fluorescent cells when the AODC method is employed. Biofilm clusters were observed in samples collected throughout the year, with single rods, short rods, and coccoid-shaped bacteria mainly observed. However, dividing cells were detectable in samples collected during the seasonal peak periods of cholera (Fig. 4B and D). Free-swimming cells and thin biofilms entrapping elongated to short rods were observed to be predominant in samples collected during the spring and fall. However, the most common morphologies of bacterial cells were short rods and coccoidal cells, mostly within thick clusters of biofilms, and rarely, elongated rods, as observed in samples collected during the winter and monsoon periods. At those times of the year, most of the bacteria were observed to occur as clusters of cells within thick biofilms (Fig. 4A and C).

**FIG 5:**
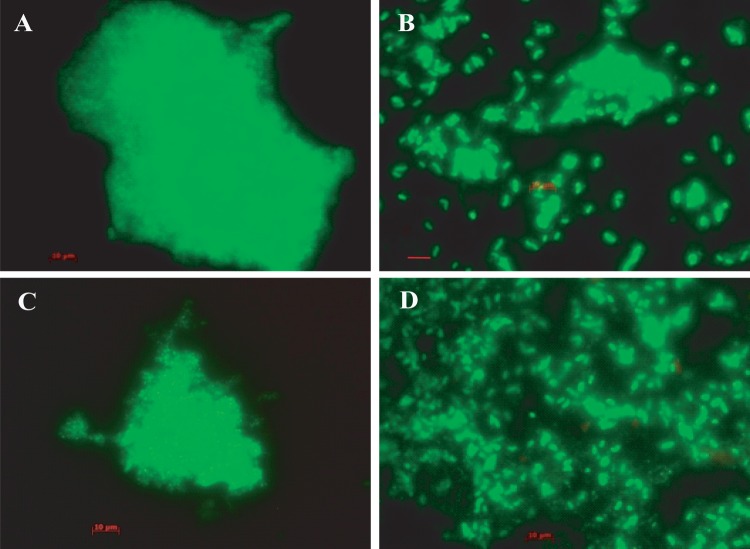
Direct fluorescent monoclonal antibody (DFA) detection of V. cholerae O1 in the estuarine aquatic ecosystem of the Bay of Bengal, Bangladesh, during different seasons of the year. (A to D) Micrographs show biofilms of V. cholerae O1 in water samples collected during winter and monsoon months (A and C) and free-living V. cholerae O1 cells in water samples collected in spring and fall months (B and D). (Scale bars in red indicate 10 µm.)

The DFA micrographs revealed that V. cholerae O1 cells were present in all samples collected throughout the year (Fig. 5A to D), an important observation. Epifluorescence microscopy showed V. cholerae O1 appearing as individual, brightly visible, free-swimming curved rods in the spring (83.33%) and fall (100%) (Table 2 and Fig. 5B and D) and in thick biofilms (clusters of cells) in samples collected during the winter (97.62%) and monsoon season (80.95%) (Table 2 and Fig. 5A and C). Actively dividing V. cholerae O1 appeared as curved rods, but most of the cells were observed to be present within thin biofilms in these samples collected during the spring and fall (Table 2 and Fig. 5B and D). V. cholerae O1 cells detected within thick clusters of biofilms were reduced in size, especially cells observed in samples collected during winter and monsoon months, when biofilm formation increased significantly (Table 2 and Fig. 5A and C).

**FIG 6:**
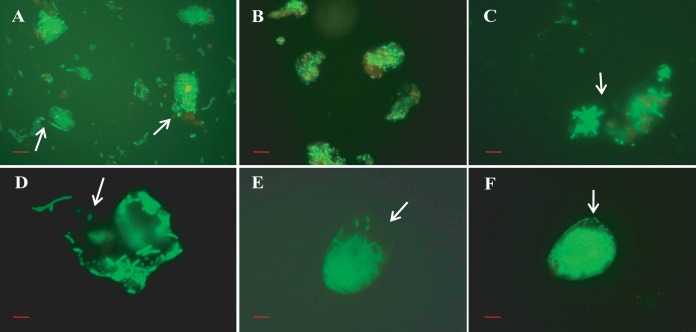
(A to F) Micrographs of acridine orange-stained microcolonies of the bacterial communities, representing stages in bacterial biofilm formation, including cell assembly (A and B) and secretion of exopolysaccharide into packages to form small consortia (C to F). Scale bars in red indicate 10 µm.

**FIG 7:**
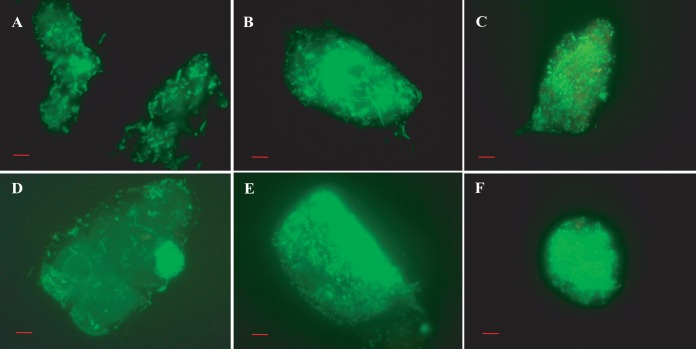
(A to F) Observation of bacterial communities forming biofilms *in situ*. Acridine orange-stained biofilm consortia occur free-floating in the aquatic environment. Scale bars in red indicate 10 µm.

Micrographs of biofilm clusters in the spring season (samples viewed under the fluorescence microscope) exhibited saclike structures containing rod-shaped bacteria. The gateway observed at one end of the saclike structure resembled the vacuoles of amoeba, as indicated by arrows in Fig. 6. Samples collected later in the spring contained larger clusters of cells, but these were compact, with smaller, coccoid cells dominant (Fig. 7). Furthermore, these water samples showed most of the bacteria in assemblages of various sizes and shapes and only rarely as free-swimming cells (Fig. 7). In samples collected during the spring months, V. cholerae O1 occurred as individual cells within a biofilm matrix, that is, cells multiplying in response to the seasonal rise in temperature, interpreted as emerging from a dormant state to assemble into thin clusters of cells (Fig. 4 and 5, panels B and D).

Compact bacterial cell assemblages were dominant in samples collected late in the autumn, when both water and air temperatures declined, proliferating during early days of winter and in late spring during monsoon rains (Fig. 4 and 5, panels A and C). In both instances, most of the free-swimming cells that appeared rod or comma shaped during the spring and fall formed clusters within biofilms, giving the assemblages of bacteria the appearance of a microconsortium (Fig. 6), i.e., a large assemblage of a diverse population (Fig. 7).

### Colonization of plankton and particles.

**FIG 8:**
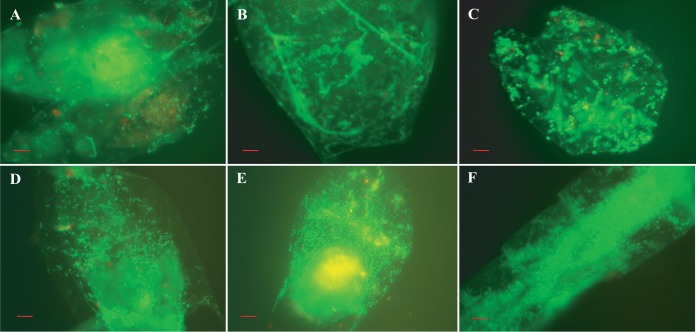
(A to F) Bacterial colonization of zooplankton in biofilms analogous to those formed by free-floating bacteria in water samples. Bacterial populations reside within or on the chitinous structures of zooplankton to form chitin-associated biofilms. (D and E) Typical biofilm formation of bacteria in association with a rotifer. Scale bars in red indicate 10 µm.

**FIG 9:**
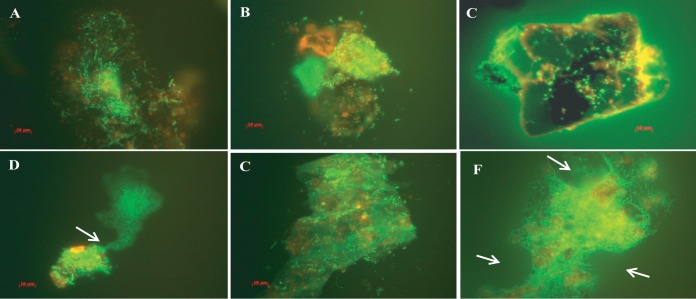
Acridine orange-stained biofilm consortia consisting of bacteria producing biofilms on both living and nonliving free-floating particulate matter. Arrows indicate circular and semicircular cavities in the biofilms. Scale bars in red indicate 10 µm.

Although quantification of bacterial cells within a biofilm was difficult because of the density of the matrix, a remarkable proportion of the naturally occurring bacteria in the aquatic environment were observed to occur in clusters of biofilms, in and on zooplankton (Fig. 8) and particulate matter (Fig. 9). Biofilms associated with particulate matter were also observed to be free floating in the water (Fig. 9). A noteworthy feature of plankton- and particle-bound biofilms was the morphological diversity of the intrabiofilm bacterial populations, showing significant variation in size and shape, characteristic of multispecies consortia. Particle-bound clusters of bacteria in biofilms were commonly observed in all samples throughout the year, suggesting colonization to be an adaptive phenomenon.

## DISCUSSION

Vibrio cholerae, the causative agent of cholera, comprises a part of the estuarine microbial community adapted to brackish water, where the bacterium is found to exist in association with zooplankton (16, 18, 19). V. cholerae flourishes under favorable environmental conditions, allowing it to be active and initiate the seasonal cycle of disease (15). Biofilm formation has been proposed as a survival strategy for V. cholerae (17), presumably to overcome adverse environmental conditions during which the bacterium exists as dormant cells in a nonculturable state, a survival strategy proposed also for many other bacteria (1, 4). The results of the study reported here confirm that only a negligible proportion of bacterial populations occurring naturally in water samples collected from estuarine villages of Mathbaria, Bangladesh, an area of cholera endemicity, can be enumerated by culturing methods (20, 21). The major finding of this study is that, in the estuarine environment, V. cholerae exists mostly as coccoid nonculturable cells within clusters of biofilms and exhibits two seasonal growth peaks of rod-shaped cells, in spring (March to May) and in autumn (September to November), corresponding to the two annual seasonal outbreaks of cholera in Bangladesh.

It has long been known that bacterial plate counts using conventional culturing methods yield only a small portion of the total number of bacteria present in environmental samples (22, 23). The general observation is that, under low-nutrient conditions in the natural environment, bacteria rarely respond to culturing methods (20, 21), as observed in the present study, suggesting limitations of this method for reflecting the bacterial most probable number. This is presumably because of the specialized requirements for media and methods for growth and capture into laboratory culture of more-fastidious bacteria, but still does not account for all that are present in water samples (1). Such culture-insensitive bacteria can be accurately enumerated by direct microscopic methods (24, 25). Acridine orange (AO) staining can visualize bacteria for enumeration by a direct microscopic method (26, 27), as AO binds to DNA and causes viable bacterial cells to appear green and, hence, differentially visible (26, 27). In the study reported here, data were obtained showing that a majority of bacterial cells in water samples collected from ponds in estuarine villages of Mathbaria, where cholera is endemic (28), fluoresced green, indicating that viable cells were present in large numbers in these samples, significantly more than were able to be cultured.

Bacterial communities in the natural environment persist by adaptively responding to environmental changes, namely, temperature, pH, and osmolarity (1). Some bacteria can modify their cellular morphology in response to environmental stimuli, as well as during the course of pathogenesis, suggesting that the change of shape is a means of adaptation (29). In this study, the observed morphological change of bacterial cells from mostly rod shaped to mostly coccoid during the onset of monsoon and immediately thereafter has adaptive implication due to osmolar change and depletion, presumably from excessive rainfall and dilution. Coccoid cells represent dormant forms of bacteria responding to unfavorable chemical or physical conditions of their environment (30). V. cholerae O1, the causative agent of cholera, a curved, rod-shaped bacterium, has previously been reported to undergo transformation to resting, spore-like coccoid (nonculturable) morphology when conditions are not conducive for growth (1, 4, 16, 31, 32). Bacterial cell aggregation and biofilm formation have been proposed as a developmental process, sharing features of the resting stage (spore formation) of Gram-positive bacteria (11, 33) and the fruiting body of Myxococcus xanthus (11, 34–36) and stalked cell of Caulobacter crescentus (11, 37–41). In this study, V. cholerae O1 cells in the Mathbaria water samples collected during interepidemic periods of the year, i.e., the winter and monsoon seasons, showed the typical coccoid morphology of viable but nonculturable (VBNC) cells in characteristic biofilms.

Nonculturability has long been proposed to be a survival strategy for V. cholerae O1 in the aquatic environment (31), but its reservoir in the aquatic environment has not been defined in detail. As shown in this study, V. cholerae O1 in the Mathbaria estuary, an established habitat of cholera bacteria (15), occurs primarily as coccoid cells during interepidemic periods, predominantly in dense clusters of biofilms. The consortia of bacteria are enveloped within a polymeric slime excreted in response to stresses, e.g., nutritional deficiency, altered temperature, and/or pH or inhibitory substances, such as antibiotics (7–10). Structured biofilm formation historically has been proposed as progressing on solid surfaces following recognition of specific or nonspecific attachment sites (7), with metabolically active bacterial cells gathering to form consortia, presumably to overcome adverse conditions of their environment (8, 9). In this study, the cellular assemblages, i.e., bacterial consortia, were observed as saclike structures containing many bacterial cells, comprising a stage in the process of biofilm formation *in situ*, and similar to vacuoles of amoeba, since bacteria can take refuge and survive within amoeba under adverse environmental conditions (42). The observed clusters of biofilms, within which curved rods, short rods, coccoid cells, and a mixture of these morphologies were observed in samples collected throughout the year, notably during and between the well-documented seasonal cholera outbreaks, are concluded to be an important reservoir for toxigenic V. cholerae O1. Decaying plankton comprises a highly likely source of particulate matter to which V. cholerae O1 can attach, as observed in the present study, in addition to its role as a commensal of chitinous fauna, namely, crabs, shrimp, and zooplankton (43–47).

Nonculturable V. cholerae in biofilms that formed in Mathbaria water microcosms were found to resume active growth in animal challenge experiments after having been nonculturable in the microcosms for more than 1 year (17). It has been proposed that preepidemic enrichment of nonculturable V. cholerae in the human host provides a method of amplification of epidemic V. cholerae immediately before the onset of an epidemic (48, 49), contributing to subsequent amplification of transmission via the fecal-oral route of human transmitted clones. Thus, the natural environmental reservoir of V. cholerae O1 can be considered to account for the consistent and persistent pattern of cholera epidemics historically documented for this disease in coastal Bangladesh (50). That is, the presence of biofilm-bound nonculturable V. cholerae O1 cells harboring the *ctxA* gene in the Mathbaria estuaries plays a significant role in the annual seasonal outbreaks of cholera in this region (28, 51).

In this study, toxigenic V. cholerae O1 occurring naturally in the coastal aquatic environment of Mathbaria, Bangladesh, could be cultured from water samples collected during spring months, when the water temperature was rising (52) and again from water samples collected in the fall. Individual cells of V. cholerae O1 were detected by culture during epidemic periods, but during interepidemic periods, they occurred mainly within biofilms, in a dormant, nonculturable state (31, 53). With warmer water and air temperatures in spring, the single cells and biofilm-bound V. cholerae O1 were clearly detectable, appearing as curved, rod-shaped cells in an actively growing, culturable state (17).

In an earlier study, plankton blooms related to rise in the sea surface temperature of the Bay of Bengal were found to be correlated with the occurrence of cholera in Bangladesh (52, 54), in two distinct seasonal peaks, before and after the annual monsoon (12–15). The existence of a V. cholerae O1 life cycle has long been debated, mainly because V. cholerae O1 is difficult to culture from natural water systems, even from samples collected during cholera epidemics (32, 55), and it is even more rarely cultivated from the environment during interepidemic periods (18). From the results of the ecological surveillance of V. cholerae O1 carried out in this study, it can be concluded that biofilms and the consortia of bacteria contained in the biofilm comprise a reservoir of toxigenic V. cholerae O1 and represent a component of an annual life cycle of this bacterium in the Bay of Bengal estuary. Recurrent seasonal epidemics of cholera in the coastal villages of Bangladesh may well be explained, at least in part, by the natural life cycle of V. cholerae, the causative agent of cholera.

## MATERIALS AND METHODS

### Collection and processing of environmental samples.

A total of 156 water and 312 plankton samples were collected biweekly from December 2005 to November 2006 from ponds in Mathbaria. Six ponds serving as drinking water sources and used for domestic purposes, such as washing utensils and bathing, were selected as sampling sites (Fig. 1). All samples were collected in sterile dark Nalgene bottles (Nalgene Nunc International, St. Louis, MO) employing aseptic technique. Samples were placed in an insulated plastic box for transport at ambient air temperature from collection sites to the central laboratory of the International Centre for Diarrhoeal Disease Research, Bangladesh (icddr,b), in Dhaka, Bangladesh (28). All samples were processed the day following collection, with approximately 20 h elapsing between sample collection in the field and processing in the laboratory (56). All samples were analyzed by acridine orange direct count (AODC) (27) to obtain total bacterial counts and heterotrophic plate counts (HPC) for total culturable bacterial counts (57–59). Direct plate counts of culturable V. cholerae O1 were obtained using thiosulfate-citrate-bile salts-sucrose agar (TCBS) (Difco, USA) (60). Samples were subjected to direct fluorescent antibody (DFA) assay for detection of V. cholerae O1 (61). Water samples were preincubated overnight with 0.025% yeast extract and 0.002% nalidixic acid and then subjected to multiplex PCR (M-PCR) (62).

### AODC.

AODC was performed as described by Hobbie et al. (27). Briefly, samples were preincubated overnight in the dark with 0.025% yeast extract (Difco Laboratories, Detroit, MI) and 0.002% nalidixic acid (Sigma). After incubation, samples were fixed with 4% formaldehyde, serially diluted 10-fold, and stained for 2 min with acridine orange (Sigma) at a 0.1% (wt/vol) final concentration. Samples were filtered using polycarbonate filters (0.2-µm pore size and 25-mm diameter; Millipore) prestained with Irgalan black dye. Stained bacteria on the membrane filters were counted using an epifluorescence microscope (Axioskop 40; Carl Zeiss, Inc., Göttingen, Germany). Total direct bacterial counts (including viable and VBNC bacteria) were averaged after counting 20 microscopic fields. Photographic records of bacteria and biofilms were captured using a digital camera (AxioCam MRc; Carl Zeiss, Inc., Göttingen, Germany) connected to the epifluorescence microscope.

### Heterotrophic plate count (HPC).

Samples were serially diluted 10-fold in phosphate-buffered saline (PBS), and 100 µl of diluted sample spread on the surface of HP agar (3 g peptone, 0.50 g soluble casein, 0.20 g K_2_HPO_4_, 0.05 g MgSO_4_, 0.001 g FeCl_2_, and 15 g agar per liter). Inoculated plates were incubated at 37°C for 72 h. After incubation, plates were observed and colonies counted to obtain total viable bacterial counts (57–59).

### Culturable counts of V. cholerae O1.

Samples were diluted 10-fold serially in PBS, and 100-µl volumes of diluted sample were spread on TCBS agar. Inoculated plates were incubated at 37°C for 24 h. After incubation, plates were observed, presumptive colonies of V. cholerae were subcultured on gelatin agar (GA) (10 g tryptone, 10 g Trypticase, 30 g gelatin, and 16 g agar per liter), and V. cholerae O1 was confirmed by slide agglutination with polyvalent anti-O1 antiserum (63). Confirmed colonies provided total viable, culturable V. cholerae O1 counts.

### DFA assay.

Direct fluorescent antibody (DFA) counting was done as described elsewhere (61). The same samples used for AODC were also subjected to DFA assay. Samples were stained using fluorescein isothiocyanate-labeled antiserum specific for V. cholerae O1 (New Horizon Diagnostic Corp., Columbia, MD, USA). Stained samples were observed using an epifluorescence microscope (Axioskop 40; Carl Zeiss, Inc., Göttingen, Germany) connected to a digital camera (AxioCam MRc; Carl Zeiss, Inc., Göttingen, Germany).

### M-PCR.

Water samples and V. cholerae O1 strains isolated from water samples were subjected to multiplex PCR (M-PCR) for detection of the V. cholerae O1-specific genes *rfbO1*, encoding surface O antigen, and *ctxA*, encoding subunit A of cholera toxin (62).

### Statistical analysis.

Environmental data collected between December 2005 and November 2006 were analyzed statistically using SPSS, Inc. (version 17.0) to assess the culturability of bacteria, including toxigenic V. cholerae O1, at various seasons of the year in the Bay of Bengal Estuary.
